# Was Terson's Tersons?

**Published:** 2012

**Authors:** Horace B Gardner

**Affiliations:** Manitou Springs, Colorado, United States

**Keywords:** Terson’s syndrome, Vitreous hemorrhage, Subarachnoid hemorrhage

## Abstract

Terson’s syndrome has had multiple definitions. The original definition is unlikely to have included Terson’s original case. An updated definition based on mechanism is proposed and related to the original case described by Terson.

## INTRODUCTION

Paunoff firstly defined Terson’s syndrome in 1962 [1] as vitreous hemorrhage in association with subarachnoid hemorrhage (SAH) and named it for a 1900 article by Terson [2]. However, Terson’s case may not have fit this definition. The definition has been modified with time, using a combination of signs to identify it. The changes in the definition and the reasons for these changes are discussed and a definition based on mechanism proposed.

## HYPOTHESIS

Syndromes are defined as a collection of signs and/or symptoms identifying a particular disease process. A disease may also be defined by it’s cause. It is suggested that Terson’s syndrome be defined in this manner, as intraocular hemorrhage caused by an abrupt increase in intracranial pressure (ICP).

## DISCUSSION

When Paunoff defined Terson’s syndrome, it is unlikely the case originally describes by Terson would have fit the definition. Although there is no doubt Terson’s case had vitreous hemorrhage, it is not at all evident it had SAH. 

Both Terson and Paunoff discuss the importance of vitreous hemorrhage in this condition, noting the possibility that such hemorrhage might arise directly from blood tracking directly from the brain through the optic nerve sheaths and into the vitreous. Even Terson thought this unlikely since pathology specimens already existed showing intracranial and vitreous hemorrhage with no hemorrhage in the optic nerve sheaths. He also thought significant optic nerve sheath hemorrhage in his case to be unlikely since his patient achieved good recovery of vision. That the vitreous hemorrhage arose instead from the retinal vasculature was also considered (and preferred) by both Terson and Paunoff. It is now generally agreed that both the subarachnoid and subdural spaces of the optic nerve “end blindly. They do not communicate with any of the intraocular chambers” [3]. Thus, although extension of intracranial hemorrhage by this route is not possible, it still persists in the modern literature. With the realization that the vitreous hemorrhage comes from the retinal vasculature, the differentiation of cases with hemorrhage in the retinal layers from those where such hemorrhage broke through the internal limiting membrane became unimportant and the definition of Terson’s syndrome was modified to include any intraocular hemorrhage. 

The importance of SAH has also undergone revision. As noted above, the original definition included SAH but it is unlikely Terson’s 1900 case had SAH since this is usually accompanied by severe headache with localizing signs appearing later while, Terson’s case awoke with hemiplegia and was more likely a cerebral vascular accident, either hemorrhagic or non-hemorrhagic. Terson thought it was hemorrhagic but admitted he could not be certain. The subarachnoid location, rather than subdural, is usually from an arterial source and might produce a more abrupt ICP elevation than a subdural bleed which is often of venous origin. However intraocular hemorrhage has been noted with subdural hemorrhage (SDH) and the definition expanded to include any intracranial hemorrhage.

Both of these changes are well demonstrated by the definitions of Terson’s syndrome in the American Academy of Ophthalmology’s Basic and Clinical Science course which in the 1984-85 edition says “Terson syndrome is vitreous hemorrhage in association with subarachnoid hemorrhage” while the 2012 definition states “Terson syndrome is recognized as vitreous and sub-ILM or subhyaloid hemorrhage caused by an abrupt intracranial hemorrhage”.

Intraocular hemorrhage also occurs with other intracranial events other than hemorrhage, such as in meningitis and crush injuries. 

Defining a condition by mechanism rather than by a collection of signs allows a more scientific analysis. Thus it is proposed Terrson’s syndrome be defined as hemorrhage from the retinal vasculature caused by an increase in intracranial pressure , just as papilledema is defined as optic nerve head swelling from imcreased ICP. Smith and Kearns [4] have produced such retinal hemorrhage in a non-human primate and Firsching [5] has shown that in the great majority of cases in humans, pressure in the intraocular portion of the central retinal vein directly reflects ICP. Thus, as suggested by Muller [6] and others, increased ICP may cause the intraocular hemorrhage seen in Terson’s syndrome. The pattern and degree of hemorrhage may reflect the time course of ICP elevation.

## CONCLUSION

Defining Terson’s syndrome by mechanism will allow conditions such as intracranial hemorrhages of any source (subarachnoid, subdural, epidural, intraventricular) as well as non-hemorrhagic conditions such as infection or cerebral edema from multiple causes to be compared. It will also insure that Terson’s original case is included.

## DISCLOSURE

The authors report no conflicts of interest in this work.
